# Supplementary material for: A composite metric for evaluating system resilience with non-idealistic performance curves
Source: PLoS One. 2025 Nov 12;20(11):e0335909. doi: 10.1371/journal.pone.0335909 (PMC12611161; doi:10.1371/journal.pone.0335909)
Supplement: S3 File — This file contains another questionnaire of the survey distributed in February 2023. The questionnaire was set up using the Forms software provided by Google®. (PDF) [file pone.0335909.s003.pdf]

© 2025 The Authors. All figures are self-generated and published under CC BY 4.0.

Resilience: performance assessment

Introduction to the performance curve

The resilience performance curve graphs the measure of system performance on a scale 0 to 1 with time. Here 0 represents total failure to perform and 1 respects adequate performance. Typically, the performance curve in literature looks like **Fig 1.**
Now for optimizing systems with a certain granularity in time steps, the system performance may look like **Fig 2.**

The x-axis represents the time and the y-axis on the performance curve represents the 'measure of performance (MOP). 'tstart' and 'tend' mark th beginning and end time of the extreme event impact.
For energy systems, MOP is the fraction of energy that is supplied to meet demand at every time step.
MOP = 1.0 means adequate supply (i.e.supply >= demand) and MOP = 0, means complete blackout. Everything in between is the fraction of system that is receiving energy supply at that time instant.

Fig1 : Typical performance curve

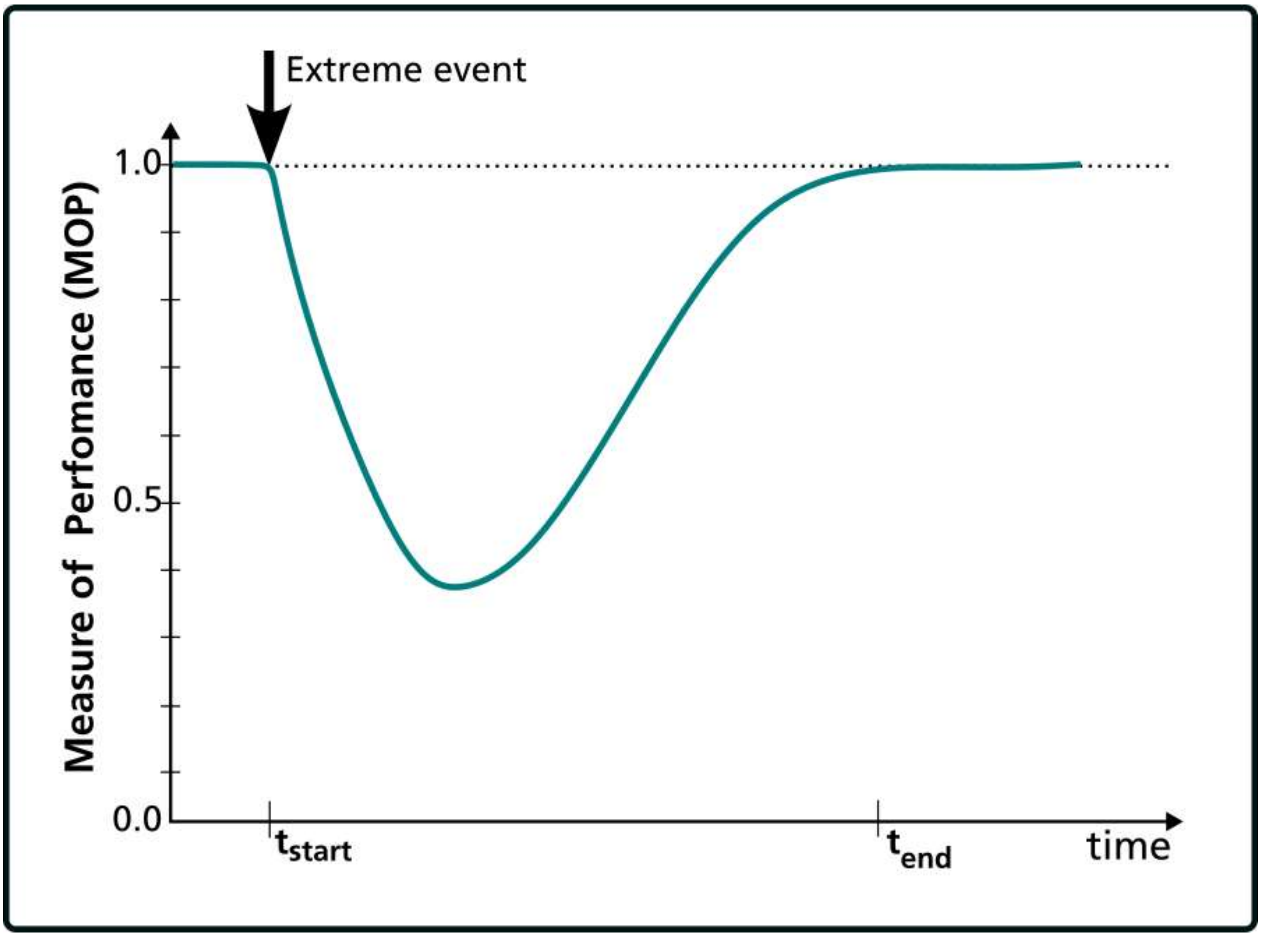

Fig2 : Example performance curve possible from energy system optimization

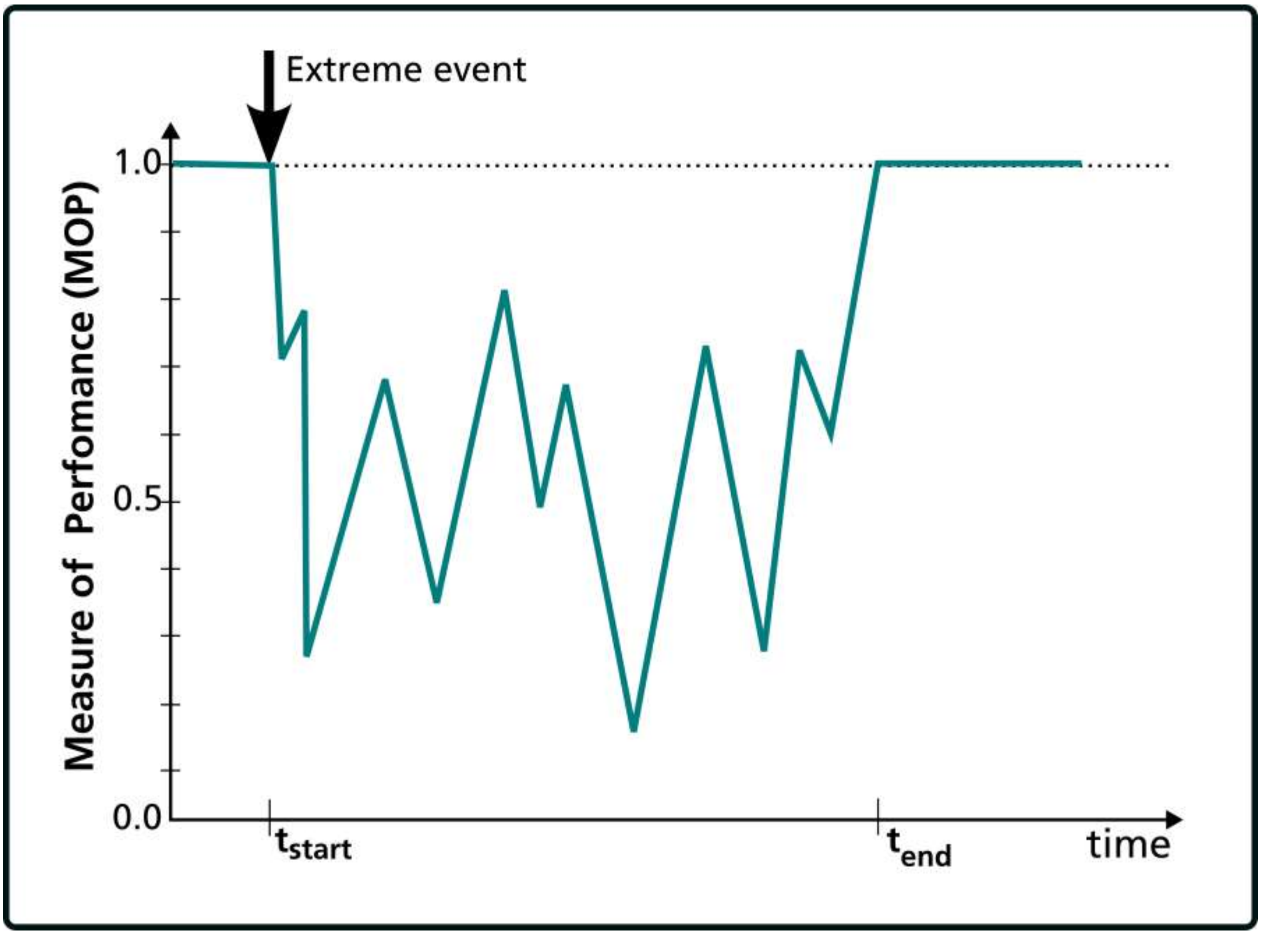

What is this survey for?

Since our performance curve does not clearly match the typical curve, standard resilience metrics don't work for us! Hence we are devising a new metric trying to capture the essence of the system's *(lack of)* performance.

This survey is a means to verify if the criteria considered in our metric matches expert opinion in the resilience and energy community .
As an energy scientist, system analyst or researcher in system resilience, you are definitely eligible and capable of answering the main questions. But for better response assessment, please answer the questions below.

(Now even if you think you are not an expert for this survey, the following page should break down a few things for you!)

1. On a scale of 1-5, how will your rate your knowledge and experience in energy system analysis ? **(1: lowest, 5 : highest)** *[5+ years of experience is already expert level for this survey]*

Mark only one oval.

	1	2	3	4	5	
	◯	◯	◯	◯	◯	

2. On a scale of 1-5, how will your rate your knowledge and experience in resilience or performance assessment of the system ? **(1: lowest, 5 : highest)** *[5+ years of experience is already expert level for this survey]*

Mark only one oval.

	1	2	3	4	5	
	◯	◯	◯	◯	◯	

Some key points!

- The domain of interest is where MOP<1. Now you may have guessed, at any time step, if MOP = 0.3, then it means that 70% of the demand is unmet at this time.
- The overall drop in system's performance over time thus accounts to the amount of un-served energy due to the extreme event.
- An important marker you will see is the **'critical measure of performance (Pcrit) '** . This is extrinsically set and it represents the minimum measure of performance necessary to keep critical infrastructure (e.g. hospitals, water-supply etc.) operating. I.e. a drop in performance below *Pcrit* will indicate failure to supply energy to some critical buildings or facilities. See *Fig 3* as an example.

Fig 3 : Example performance curve with critical measure of performance Pcrit = 0.5

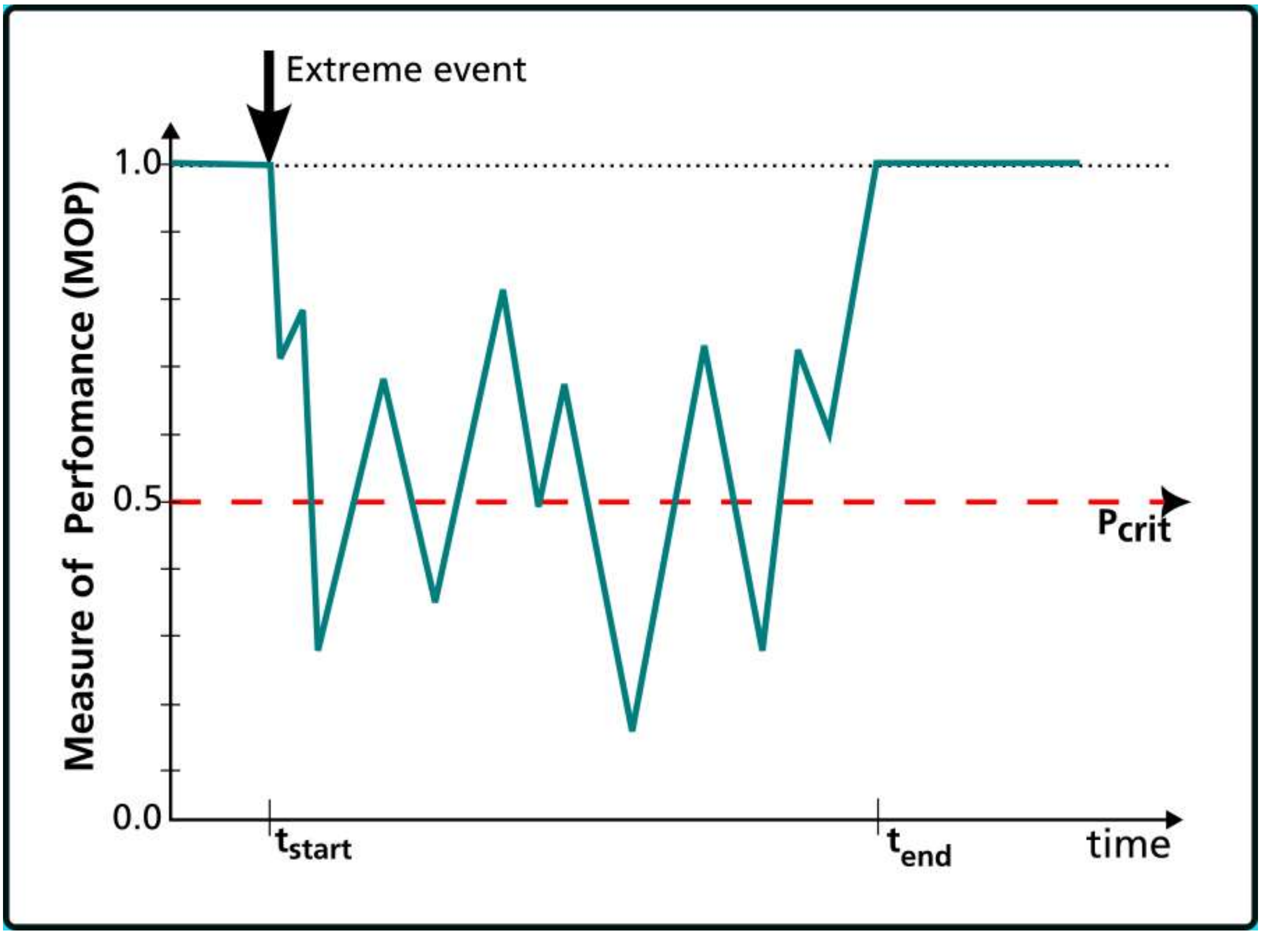

Gearing up!

Note that throughout this survey, the extreme event causing the impact, the time duration of impact and value of critical measure of performance *(Pcrit)* for the system are fixed and same for every question.

(Remember, there is always one question : ***Which is worse?****)*

All set, let's begin! Are you ready?

Questions : the real part!

3. Q1. Which of the two curves shows **worse** performance than the other?

Mark only one oval.

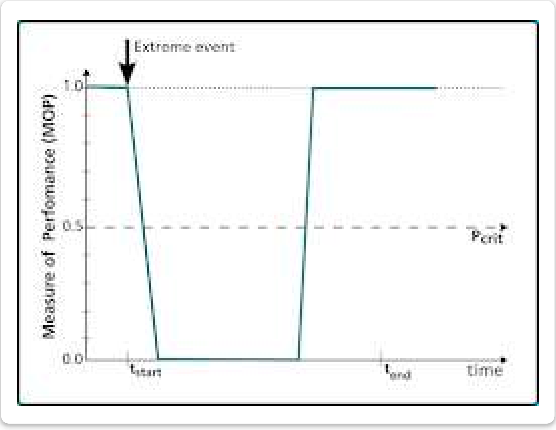

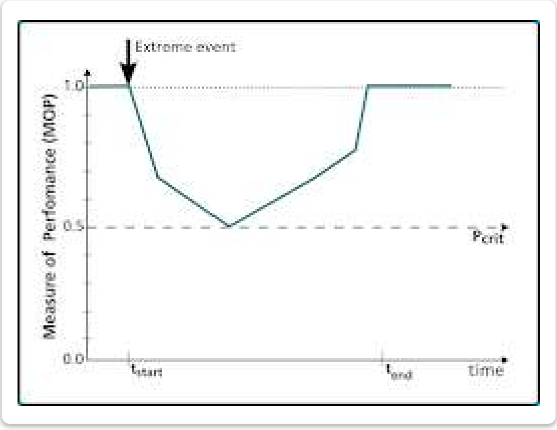

- A
- B

4. Q2. Which of the two curves shows **worse** performance than the other?

Mark only one oval.

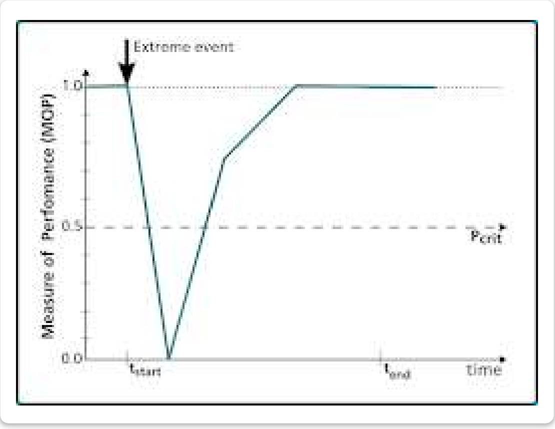

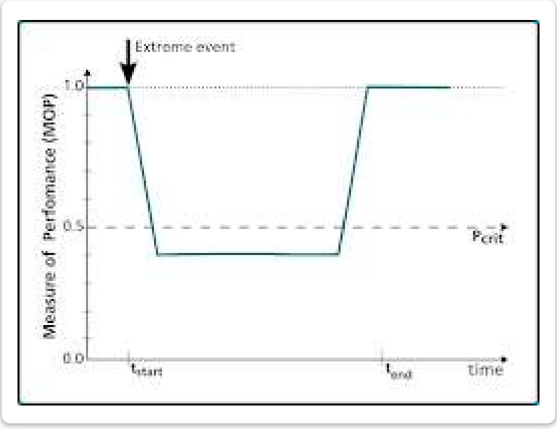

- A
- B

5. Q3. Which of the two curves shows **worse** performance than the other?

Mark only one oval.

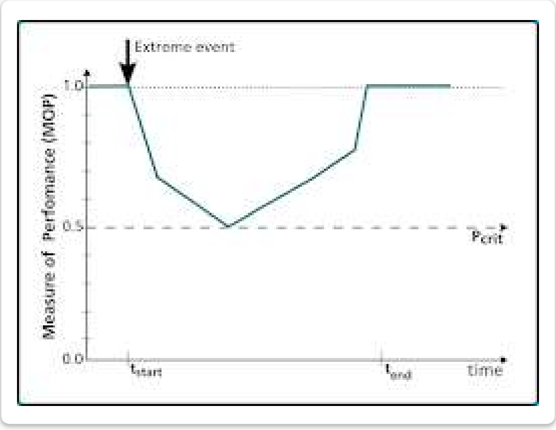

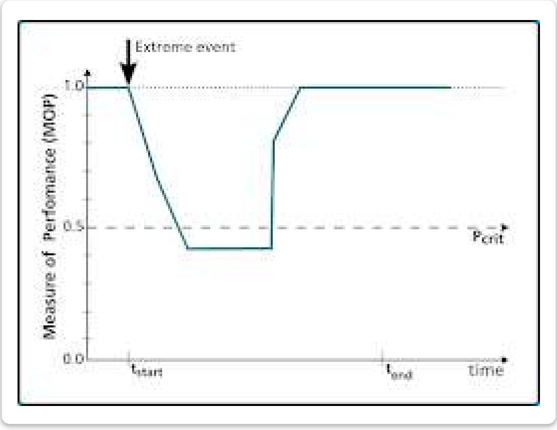

- A
- B

6. Q4. Which of the two curves shows **worse** performance than the other?

Mark only one oval.

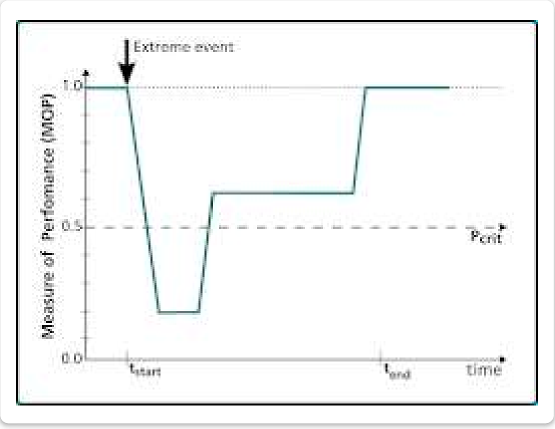

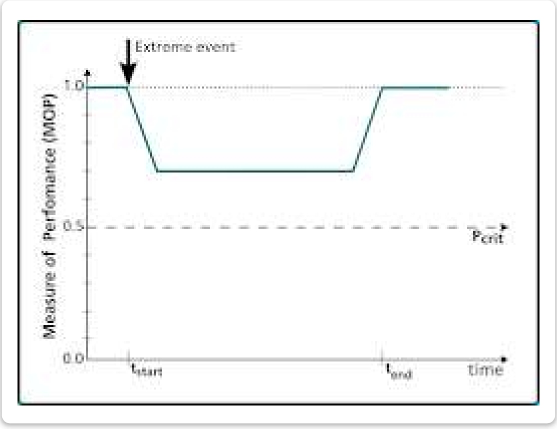

- A
- B

7. Q5. Which of the two curves shows **worse** performance than the other?

Mark only one oval.

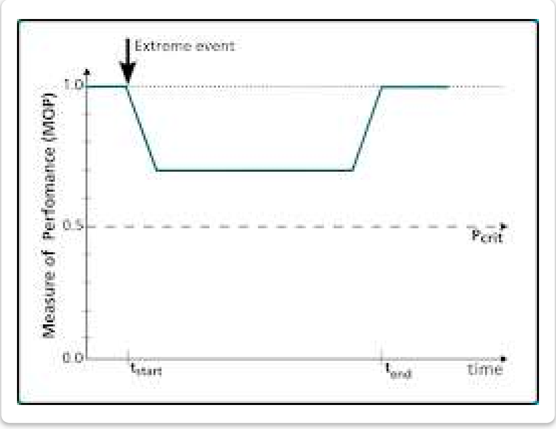

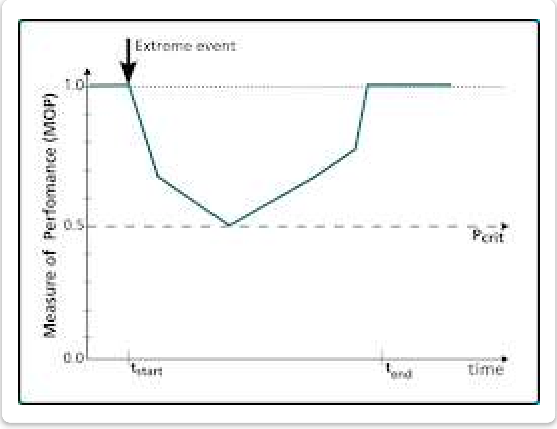

A

B

8. Q6. Which of the two curves shows **worse** performance than the other?

Mark only one oval.

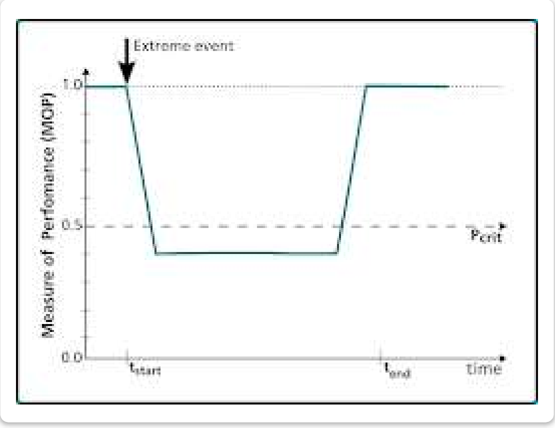

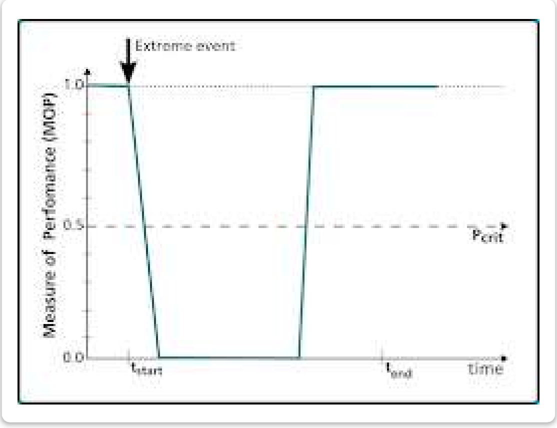

A

B

9. Q7. Which of the two curves shows **worse** performance than the other?

Mark only one oval.

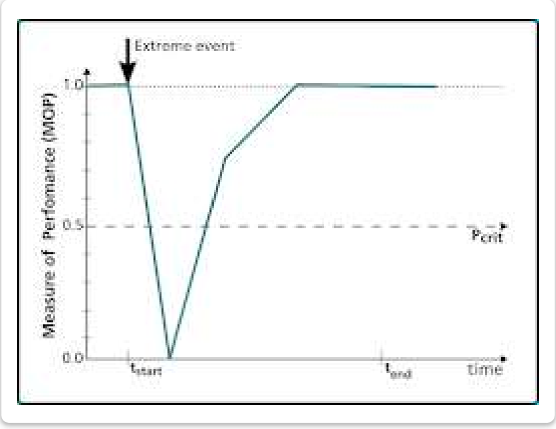

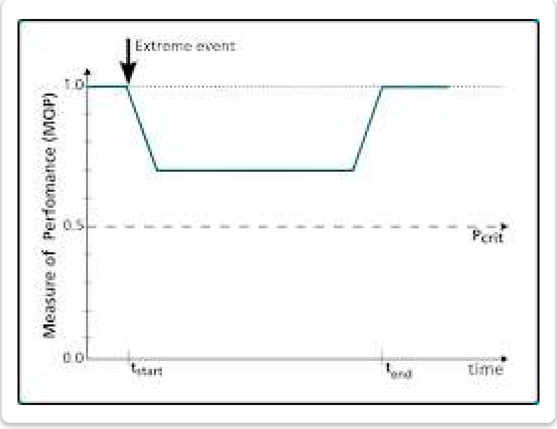

A

B

10. Q8. Which of the two curves shows **worse** performance than the other?

Mark only one oval.

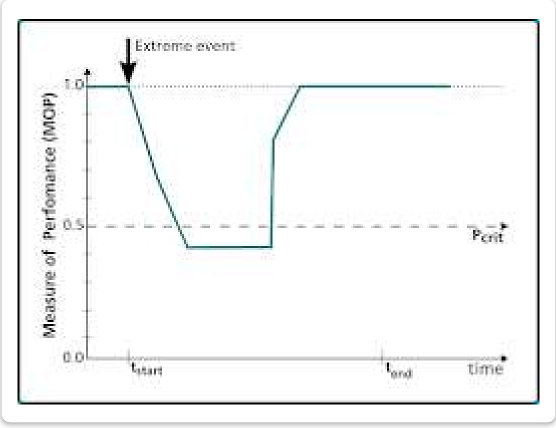

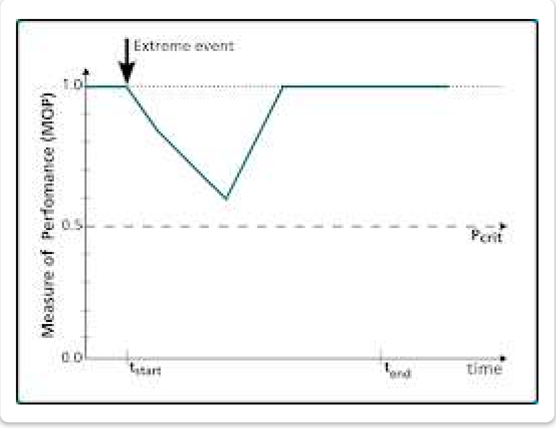

A

B

11. Q9. Which of the two curves shows **worse** performance than the other?

Mark only one oval.

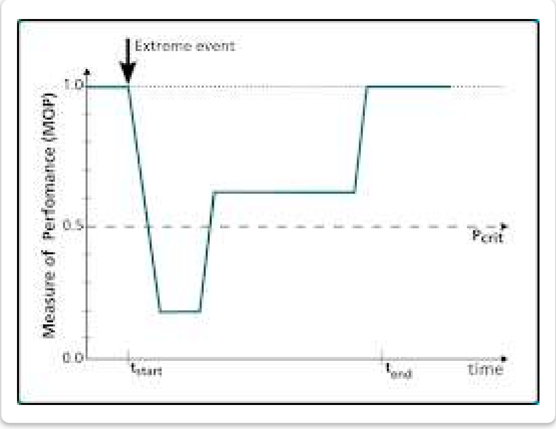

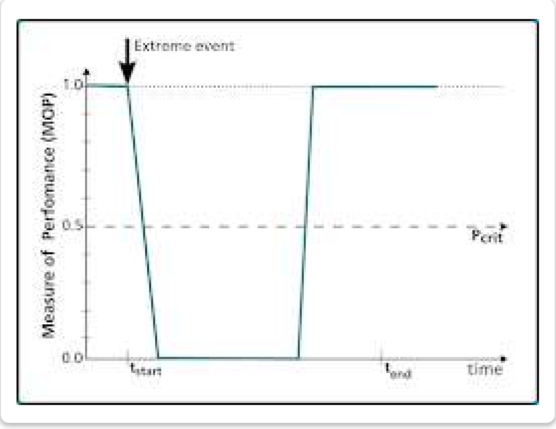

- [ ] A
- [ ] B

12. Q10. Which of the two curves shows **worse** performance than the other?

Mark only one oval.

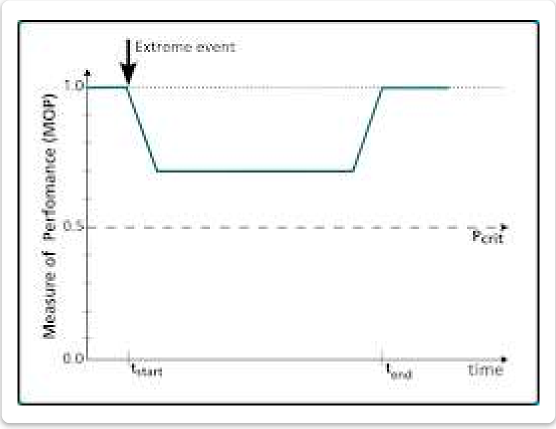

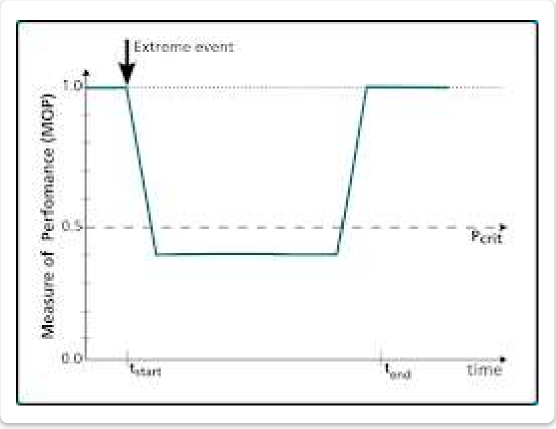

- [ ] A
- [ ] B

13. Q11. Which of the two curves shows **worse** performance than the other?

Mark only one oval.

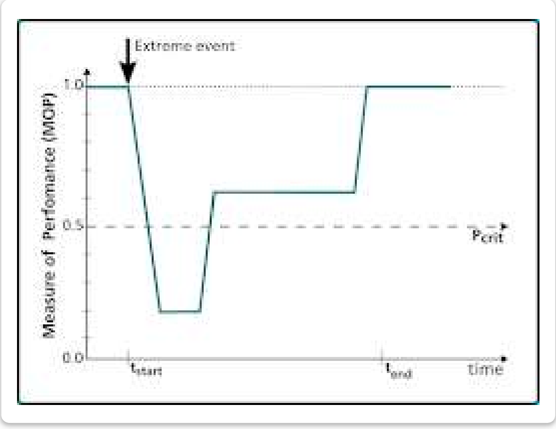

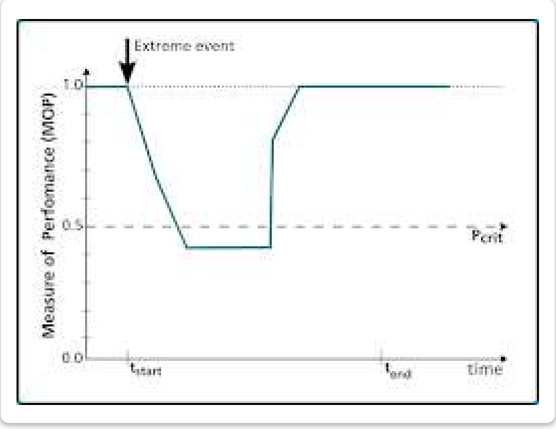

A

B

14. Q12. Which of the two curves shows **worse** performance than the other?

Mark only one oval.

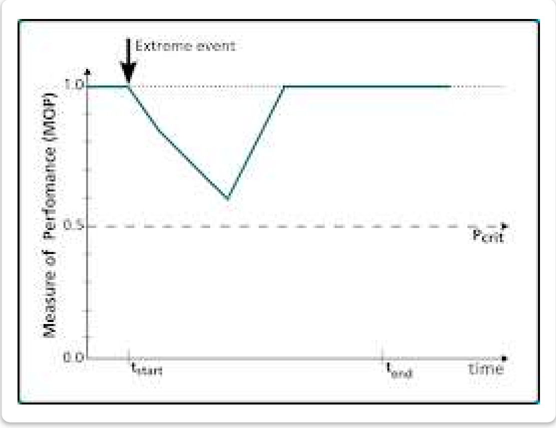

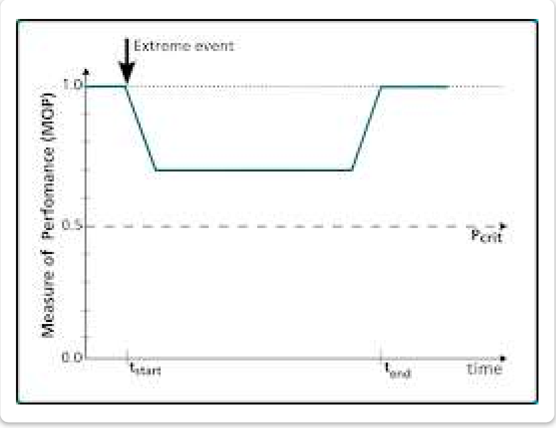

A

B

15. Q13. Which of the two curves shows **worse** performance than the other?

Mark only one oval.

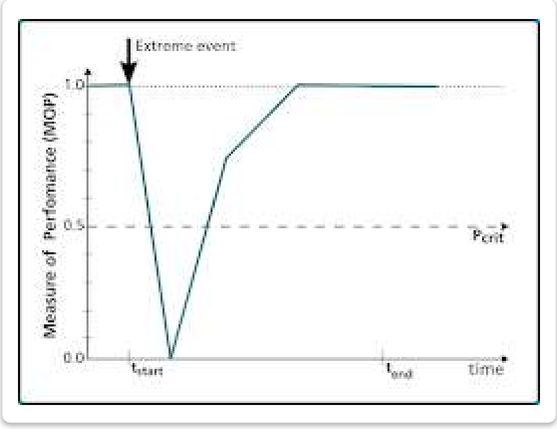

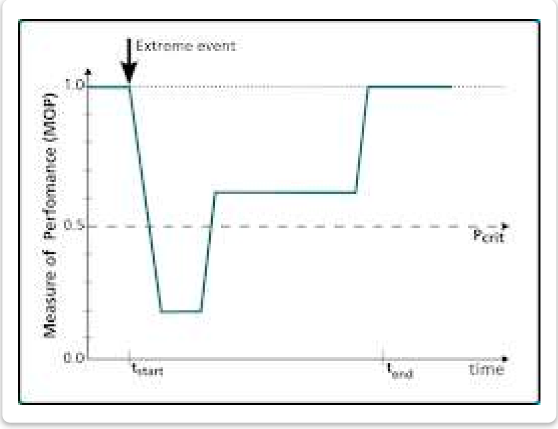

- [ ] A
- [ ] B

16. Q14. Which of the two curves shows **worse** performance than the other?

Mark only one oval.

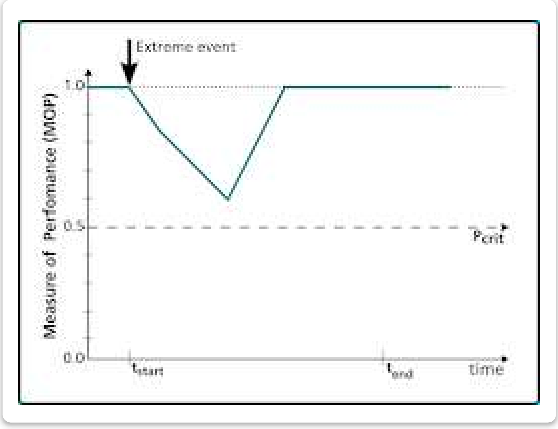

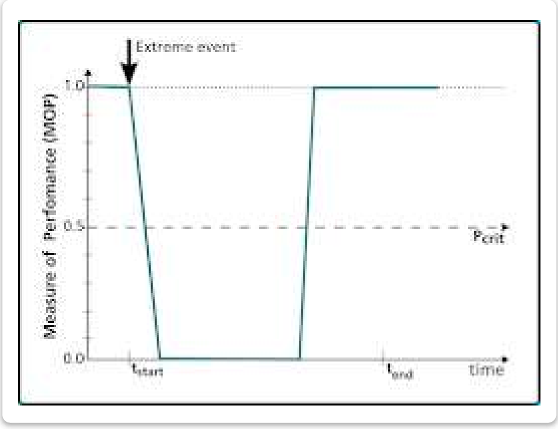

- [ ] A
- [ ] B

This content is neither created nor endorsed by Google.

Google Forms
