# Supplementary material for: A composite metric for evaluating system resilience with non-idealistic performance curves
Source: PLoS One. 2025 Nov 12;20(11):e0335909. doi: 10.1371/journal.pone.0335909 (PMC12611161; doi:10.1371/journal.pone.0335909)
Supplement: Attachment — Submitted filename: Response_to_reviewers.pdf [file pone.0335909.s006.pdf]

Response to reviewers

Reviewer #1: Resilient system design is crucial in engineering, requiring quantitative resilience assessment through various metrics. Traditional metrics depict system responses to shocks as inverse bell-shaped, triangular, or trapezoidal curves, defining disruption and restoration phases. However, these models fail for irregular responses. This paper introduces a composite metric that integrates diverse system performance curves and a user-defined threshold. Validation through expert surveys confirms its effectiveness, and benchmarking highlights its adaptability across fields with non-ideal response behaviors.

Thank you for your response and your time to review. Your summary of the submitted manuscript aligns perfectly to our aim and has assured us that the manuscript delivers the message as intended.

Reviewer #2: I would recommend the following suggestions
I would recommend to format the manuscript according to the journal format/ guidelines. For that checkout recent Plos One Publications.

Thank you for the meticulous remarks on text, table and figures formatting. We have carefully evaluated the requirements from the following sources taken from the journal website:

A. Submission Guidelines | PLOS One
B. LaTeX | PLOS One
C. PLOS Affiliations Formatting Guidelines.pdf
D. PLOSOne_formatting_sample_main_body.pdf

Please note that the submitted manuscript is built using the LaTeX software from the template linked in [B]. There are differences in some of the guidelines and the LaTeX template [B]. So, we referred to recently published papers from PLOS One also. Most changes now conform to the guidelines from sources A, C and D. However, wherever it was not clear, the style and formatting already available in the LaTeX template [B] have been maintained. When the manuscript is accepted, the entire LaTeX project will be provided to the Editorial team and formatting edits can be made before the publication if required. Please find our responses to specific comments below:

Line spacing and line numbers should be according to journal format.

- As indicated in the PLOS One guidelines [A], the manuscript is changed to double-spacing.
- The line-numbering is different in [C] and the LaTeX template [see pdf E]. Besides, the only instructions on the website [A] indicate the need for continuous linear line numbering. Additionally, line numbering is not present in published papers of PLOS One. Since, the LaTeX template style fits the requirement from the website and it may only be relevant in the review process, the numbering style is maintained as it is without changes.

In-text References and End bibliography should be rearranged accordingly as per journal's guidelines.

- We checked this and made corrections where necessary. The references and bibliography now match the style specified in [A, B, D].

Figures and tables must be arranged as per journal's format.

Thank you for the feedback, we made the following checks and alterations:

- Captions have been changed from Figure xx. to Fig xx. and references have been changed from Fig. xx to Fig xx.
- The figure names, labels and captions are homogenized such that for Figx.tiff, it is labelled Figx and caption states *Fig x. Caption title*
- Text in all figures is changed to Arial font of size 10-12 as suitable
- The resolution of all figures except Fig 4, 11 and 14 is over 300dpi. For the mentioned three figures, the resolution was reduced so that the file size is below 10 MB. If uploading figure files over 10MB is allowed, a higher resolution of these images can be provided when required.
- Table formatting is checked and it presently follows the format of the LaTeX template [B]. Headings were made bold in Table 2.

Figures legends should be right after the paragraph they are discussed in.

- This has been checked and corrected in the current revision. Thank you for the remark.

Reformat paragraph alignment.

- Thank you for the hint. We noticed that, similar to line-numbering, the paragraph alignment format is different in the source [D] and the LaTeX template [B]. Since there is no specific guideline for this on the journal website, we also referred recently published papers. The format in these papers matches the LaTeX format. Hence, the paragraph alignment is maintained as it is. However, it is possible to change this if the editorial team requires it so before the publication directly in the LaTeX repository.

All supporting information should be provided in separate, Docx file.

- Thank you for this very important remark. We referred to the guidelines [A, B, D] again and realised that the supporting information we provided is rather intended as an appendix to the main body of the paper. In addition, the supplementary material provided should be called 'Supporting Information' and be mentioned in the main manuscript. We modified this entire section such that:
  - Information closely linked to the understanding of our study is grouped into a new section called *Appendix*. This is intended for additional reading, since including it within the main sections of the paper would be incoherent or divergent to the context. Yet, it should be present in the manuscript allowing readers to quickly cross-refer across sections and enhancing understanding and readability.
  - Other data and information required for transparency and reproducibility of the study are classified into *Supporting Information.* The captions are included in the manuscript and the files are provided separately
- We noted from the guidelines [D] that any file format for the Supporting Information is acceptable. Therefore, we provided .pdf and csv. that are readable on multiple platforms.

Response to Editorial team

Please note that all changes made are described meticulously in the cover letter. In the following, individual points are addressed for more clarity.

1.Please ensure that your manuscript meets PLOS ONE's style requirements, including those for file naming. The PLOS ONE style templates can be found at

https://journals.plos.org/plosone/s/file?id=wjVg/PLOSOne_formatting_sample_main_body.pdf and

https://journals.plos.org/plosone/s/file?id=ba62/PLOSOne_formatting_sample_title_authors_affiliations.pdf

Thank you for linking me to respective guidelines, they have been referred for changes and also linked above (c.f. C. and D)

2. Please note that funding information should not appear in any section or other areas of your manuscript. We will only publish funding information present in the Funding Statement section of the online submission form. Please remove any funding-related text from the manuscript.

The funding information has been removed from the Acknowledgement section.

3. Thank you for stating the following financial disclosure:

"The research in this project was sponsored through the project 'ReMo-Digital' funded by the German Federal Ministry for Economic Affairs and Climate Action (BMWK) under grant number 03EI1020B, supporting the authors Madhura Yeligeti and Hans Christian Gils. The scientific contributions of Wolfgang Nowak are supported by the Stuttgart Center for Simulation Science (SimTech)"

Please state what role the funders took in the study. If the funders had no role, please state: "The funders had no role in study design, data collection and analysis, decision to publish, or preparation of the manuscript."

If this statement is not correct you must amend it as needed.

Please include this amended Role of Funder statement in your cover letter; we will change the online submission form on your behalf.

Thank you for the hint. We would like to maintain **that the funders had no role.** We have tried to edit the financial statement during the submission. Please check it.

4. We note that you have indicated that there are restrictions to data sharing for this study. PLOS only allows data to be available upon request if there are legal or ethical restrictions on sharing data publicly. For more information on unacceptable data access restrictions, please see http://journals.plos.org/plosone/s/data-availability#loc-unacceptable-data-access-restrictions.

Before we proceed with your manuscript, please address the following prompts:

a) If there are ethical or legal restrictions on sharing a de-identified data set, please explain them in detail (e.g., data contain potentially identifying or sensitive patient information, data are owned by a third-party organization, etc.) and who has imposed them (e.g., a Research Ethics Committee or Institutional Review Board, etc.). Please also provide contact information for a data access committee, ethics committee, or other institutional body to which data requests may be sent.

b) If there are no restrictions, please upload the minimal anonymized data set necessary to replicate your study findings to a stable, public repository and provide us with the relevant URLs, DOIs, or accession numbers. For a list of recommended repositories, please see

https://journals.plos.org/plosone/s/recommended-repositories. You also have the option of uploading the data as Supporting Information files, but we would recommend depositing data directly to a data repository if possible.

We will update your Data Availability statement on your behalf to reflect the information you provide.

Thank you once again for helping us evaluate our data sharing possibilities. Since there are no ethical or legal restrictions in our case, we have concluded that we can make all the data available that is required for complete transparency and reproducibility. This is done in the form of supporting information. Please update the 'Data Availability' statement reflects that 'All data is openly available as supporting information'

5. Please include your full ethics statement in the 'Methods' section of your manuscript file. In your statement, please include the full name of the IRB or ethics committee who approved or waived your study, as well as whether or not you obtained informed written or verbal consent. If consent was waived for your study, please include this information in your statement as well.

Yes, a new subsection for this purpose is created and all the suggested information is included in the revised version of the manuscript.

6. Please review your reference list to ensure that it is complete and correct. If you have cited papers that have been retracted, please include the rationale for doing so in the manuscript text, or remove these references and replace them with relevant current references. Any changes to the reference list should be mentioned in the rebuttal letter that accompanies your revised manuscript. If you need to cite a retracted article, indicate the article's retracted status in the References list and also include a citation and full reference for the retraction notice.

Thank you for the hint. The only change made is a paper which was under review when the manuscript was first submitted, but is now published. This is highlighted in the pdf of revised manuscript with tracked changes.
