# Supplementary material for: A composite metric for evaluating system resilience with non-idealistic performance curves
Source: PLoS One. 2025 Nov 12;20(11):e0335909. doi: 10.1371/journal.pone.0335909 (PMC12611161; doi:10.1371/journal.pone.0335909)
Supplement: Attachment — Submitted filename: Response_to_reviewers_10_25.pdf [file pone.0335909.s007.pdf]

Response to reviewers

We sincerely thank all the reviewers for investing their time and for all the remarks and suggestions. They have provided us new perspectives and helped us improve the manuscript in multiple ways. In the following, we refer to all the comments we received and finally, we summarize other changes made to the document not directly linked to any specific comment.

Reviewer #1: (No Response)

Reviewer #2: All of my comments have been addressed. Therefore, I suggest to accept this manuscript.
An amazing piece of work by authors. Keep it up and Good luck.

Reviewer #5: The author provided a satisfactory reply to my feedback, and based on that, they made thorough revisions to the article.

- To Reviewers #1, #2, #5, we thank you once again for your feedback during past revisions and your support.

Reviewer #3: The authors explain that survey data are available upon request, this does not fully align with PLOS ONE's data availability policy, which requires unrestricted public access to underlying data. The authors are encouraged to deposit anonymized survey data in a public repository or include it in the supplementary materials to fulfill this requirement.

- Thank you for the feedback. While this was the case earlier, we updated the manuscript already in the previous revision and all the data has been made available as supporting information. We tried to emphasize again it in the data availability statement during submission. However, if you still find words 'survey data are available upon request' anywhere in the submission, it is representing an older stance and has not been updated. Please let us know where you see it so we can update it.

Reviewer #4: The manuscript presents a novel composite resilience metric addressing limitations of existing methods, particularly for non-idealized system performance curves. The work is theoretically sound, methodologically rigorous, and well-supported by a survey and benchmarking. However, revisions are needed to enhance clarity, justify assumptions, and strengthen the discussion.

- Thank you for your concise summary. It has reassured us that our message in the paper is understandable. With regards to all your comments, we they made sense to us and we appreciate your inputs. So, we have evaluated and modified our manuscript to address them in the following ways:

1-The composite metric fills a clear gap in resilience quantification for atypical performance curves. However, the introduction should better contextualize how this work advances beyond prior summaries.

- We modified the summary of literature in the introduction (lines 92-101) to highlight the status-quo and the gaps in the studies we reviewed. To this, we added another paragraph (lines 102-110) specifically focussing on the main contributions of our work. In our perspective, these two modifications explicitly lay down the scope of our work and provide context to the value added by our study.

2-The survey's sample size (41 respondents) is modest. Discuss potential biases (e.g., dominance of energy system researchers) and generalizability.

- Thank you for this thoughtful remark. The bias due to the small sample size is addressed using bootstrapping (refer sections 2.5.2 (lines 357-393) and 3.1 (lines 468-507)). We recognize the bias due to the predominance of energy system expertise in the survey-takers group. However, it is not feasible to expand the experiment to other scientific communities in a feasible and practical manner at this stage. Hence, we accept the possible bias and state it explicitly in lines 501-507 of the manuscript. This enhances the importance of benchmarking for metric verification, in addition to the survey.

3-Update citations to include 2023–2024 literature on resilience metrics (e.g., Raoufi et al. 2023).

- This is an important point to keep this study relevant, thank you for mentioning it. We have now updated our literature to include studies upto 2025. This includes, newer resilience metrics, literature reviews, and also studies based on empirical examples for non-typical performance curves. These references are highlighted in the manuscript in the Bibliography and also in the Appendix A1. They are also mentioned below.
- With regard to the citation you mentioned, we could not find Raoufi et al. 2023. But with some investigation, we found that Raoufi et al. 2021 is quite relevant to our work and we have added this citation.

4-Emphasize the metric's adaptability to other domains (e.g., healthcare, transport) in the conclusion.

- Thank you for this comment. We have addressed it in two ways:
    - In the introduction, we had already provided the context of complex systems where atypical performance measures can appear. Now, we added two citations that show examples of such non-typical behaviour based on empirical data, one in transmission network systems and another in social science (macroeconomics-employment payroll impact)
    - In the conclusions, we hint at complex systems in lines 603-605. Further, in lines 608-613, we state that the only prerequisite for applying the metric is a normalized performance measure and specify examples of what such measures can be in other fields.

Summary of revisions

Overall, we modified the introduction and conclusions to address the comments from reviewers. We added *13 new citations* mostly from 2024-2025 and two of the existing citations were modified since we found errors in the citation links. Additionally, we made minor language-related edits in some other sections as appropriate. All the changes made have been highlighted in the revised manuscript. They are summarized here:

1. **Two existing citations were corrected:**

    a. *(Marriott P. Journal of the Royal Statistical Society Series A (Statistics in*

Society). 1995;158(2):347–347) has been replaced by
(Efron B, Tibshirani RJ. An Introduction to the Bootstrap. 0th ed. Chapman and Hall/CRC; 1994.) since the previous citation referenced not the book itself but a commentary on the book. This is citation [80] in the references section.

b. *(Colomer J. Electoral Systems-Ramon Llull: From Ars Electionis to Social Choice Theory. 2012;doi:10.1007/s00355-011-0598-2*) corrected to *(Colomer JM. Ramon Llull: from 'Ars electionis' to social choice theory. Social Choice and Welfare. 2013;40(2):317–328. doi:10.1007/s00355-011-0598-2.)* since the previous citation title did not match the DOI correctly. This is citation [76] in the references section.

2. 13 new citations have been added: [in brackets are citation number according to manuscript]

a. [5] Laino AS, Wooding B, Soudjani S, Davenport RJ. A logic-based resilience metric for water resource recovery facilities. Environmental Science-Water Research & Technology. 2025;11(2). doi:10.1039/d4ew00649f.
b. [9] Sanabria-Fernandez JA, Alday JG. Marine protection enhances the resilience of biological communities on temperate rocky reefs. Aquatic Conservation-Marine and Freshwater Ecosystems. 2024;34(2). doi:10.1002/aqc.4101.
c. [15] Sapkota A, Karki R. Resilience Investment Against Extreme Weather Events Considering Critical Load Points in an Active Microgrid. Applied Sciences. 2025;15(13):6973. doi:10.3390/app15136973.
d. [26] Abantao GA, Ibanez JA, Bundoc PEDC, Blas LLF, Penisa XN, Esparcia Jr EA, et al. Reconceptualizing Reliability Indices as Metrics to Quantify Power Distribution System Resilience. Energies. 2024;17(8). doi:10.3390/en17081909.
e. [27] Dobson I, Ekisheva S. How Long is a Resilience Event in a Transmission System?: Metrics and Models Driven by Utility Data. Ieee Transactions on Power Systems. 2024;39(2). doi:10.1109/TPWRS.2023.3292328.
f. [36] Sajwan S, Ketan Panigrahi B, Srivastava AK. Multi-Stage Operational Resilience Metric-Driven Optimal Service Restoration in DER-Rich Power Distribution Systems. Ieee Access. 2025;13:95275–95287. doi:10.1109/ACCESS.2025.3574480.
g. [37] Toumasis N, Simms D, Rust W, Harris J, White JR, Zawadzka J, et al. Emerging resilience metrics in an intensely managed ecological system. Ecological Engineering. 2024;200. doi:10.1016/j.ecoleng.2023.107151.
h. [50] Hu T, Zong Y, Lu N, Jiang B. Dynamic Recovery and a Resilience Metric for UAV Swarms Under Attack. Drones. 2025;9(8):589. doi:10.3390/drones9080589.
i. [51] Raoufi H, Vahidinasab V. Power system resilience assessment considering critical
infrastructure resilience approaches and government policymaker criteria. IET Generation, Transmission & Distribution. 2021;15(20):2819–2834. doi:10.1049/gtd2.12218.
j. [53] Nejati Amiri MH, Gu´eniat F. Towards a framework for measurements of power systems resiliency: Comprehensive review and development of graph and vector-based resilience metrics. Sustainable Cities and Society. 2024;109. doi:10.1016/j.scs.2024.105517.
k. [61] Bruckler M, Wietschel L, Messmann L, Thorenz A, Tuma A. Review of metrics to assess resilience capacities and actions for supply chain resilience. Computers

& Industrial Engineering. 2024;192. doi:10.1016/j.cie.2024.110176

l. [66] Wachtel A, Gunda T, Caskey S, Cooper R, Womack T, Bonney K, et al. pyRoCS: A Python package to evaluate the resilience of complex systems. SoftwareX. 2025;29:101977. doi:10.1016/j.softx.2024.101977.
m. [69] Silva P, Hidalgo M, Hotchkiss M, Dharmasena L, Linkov I, Fiondella L. Predictive Resilience Modeling Using Statistical Regression Methods. Mathematics. 2024;12(15). doi:10.3390/math12152380.

3. Changes corresponding to Reviewer #4's comments:

a. *Introduction:* Lines (39-42, 54-61, 96-110) ; new citations on lines (20, 23, 26, 28, 32)
b. *Results and Discussion*: Lines (501-507)
c. *Conclusions:* Lines (602-622)
d. *Appendix:* (new citations: Appendix A1) line 633, table 2

4. Minor language-related edits (typos, avoiding verbosity, grammar etc.):

a. *Introduction:* Lines (80, 81, 85)
b. *Methods:* Lines (399, 405, 412)
c. *Results and Discussion:* Lines (579-580)
